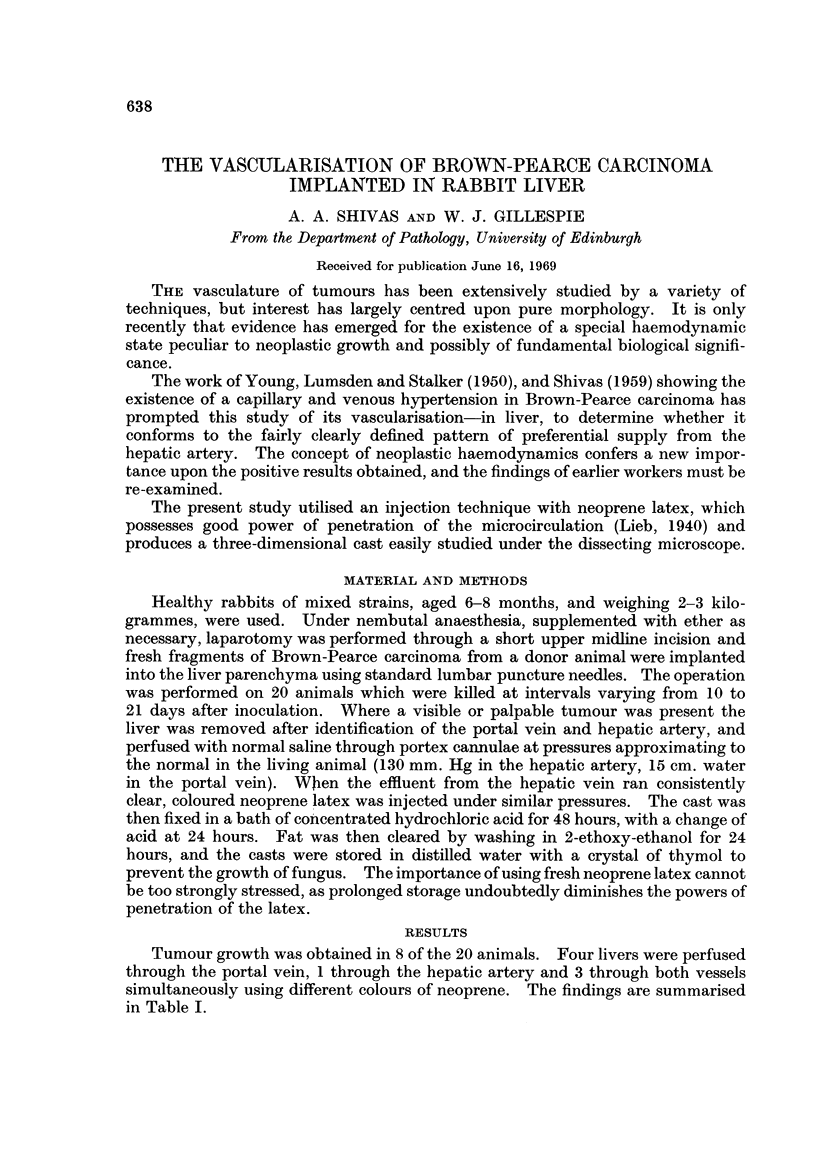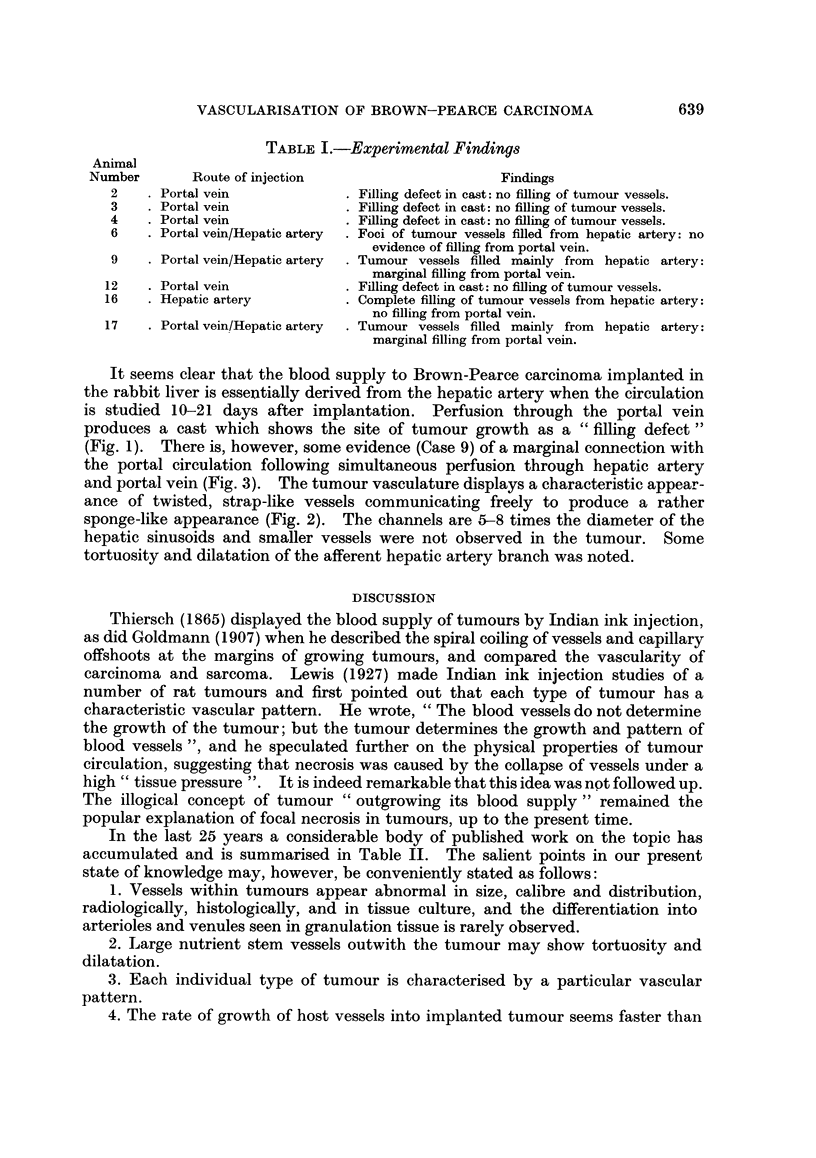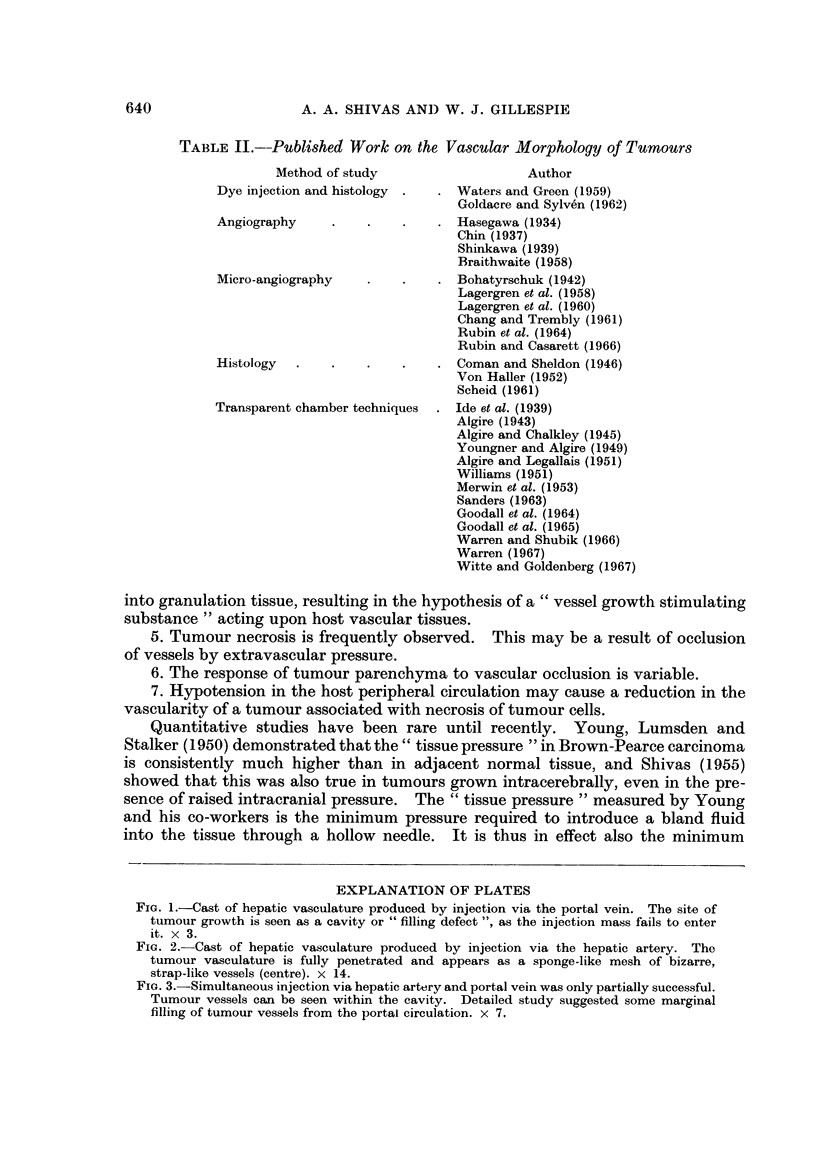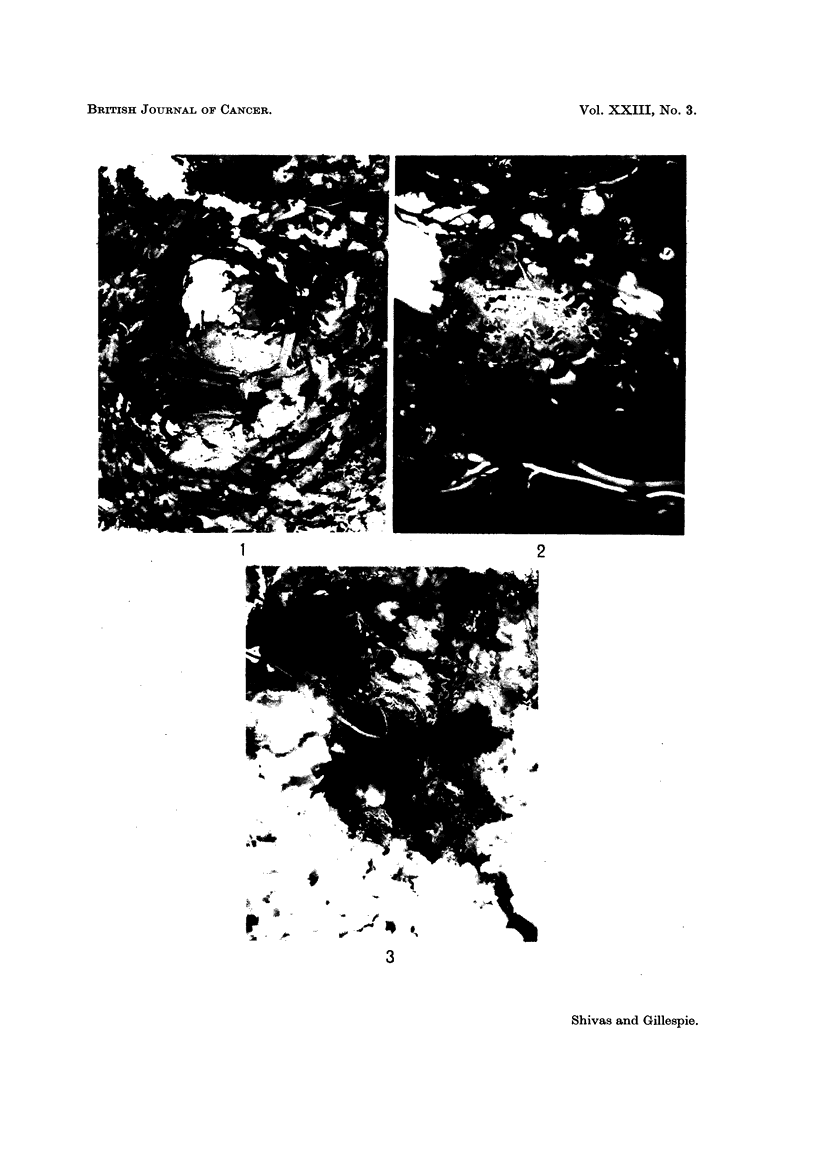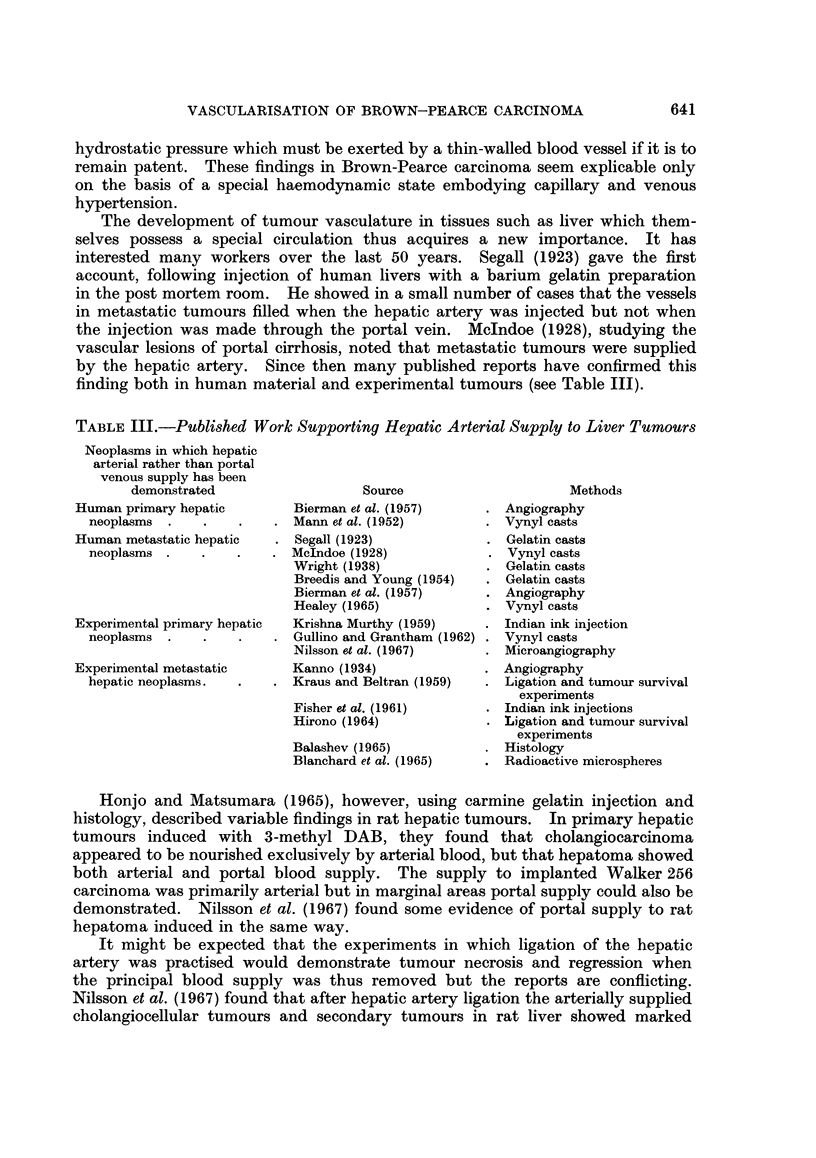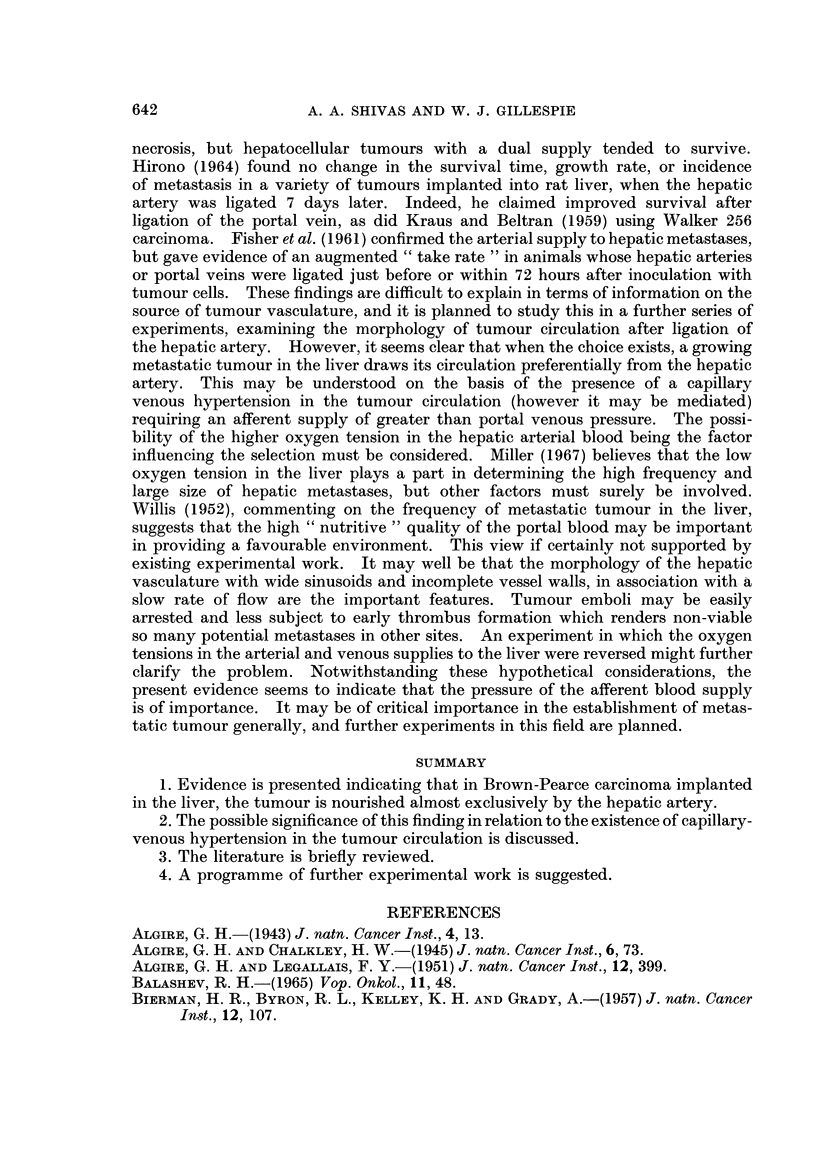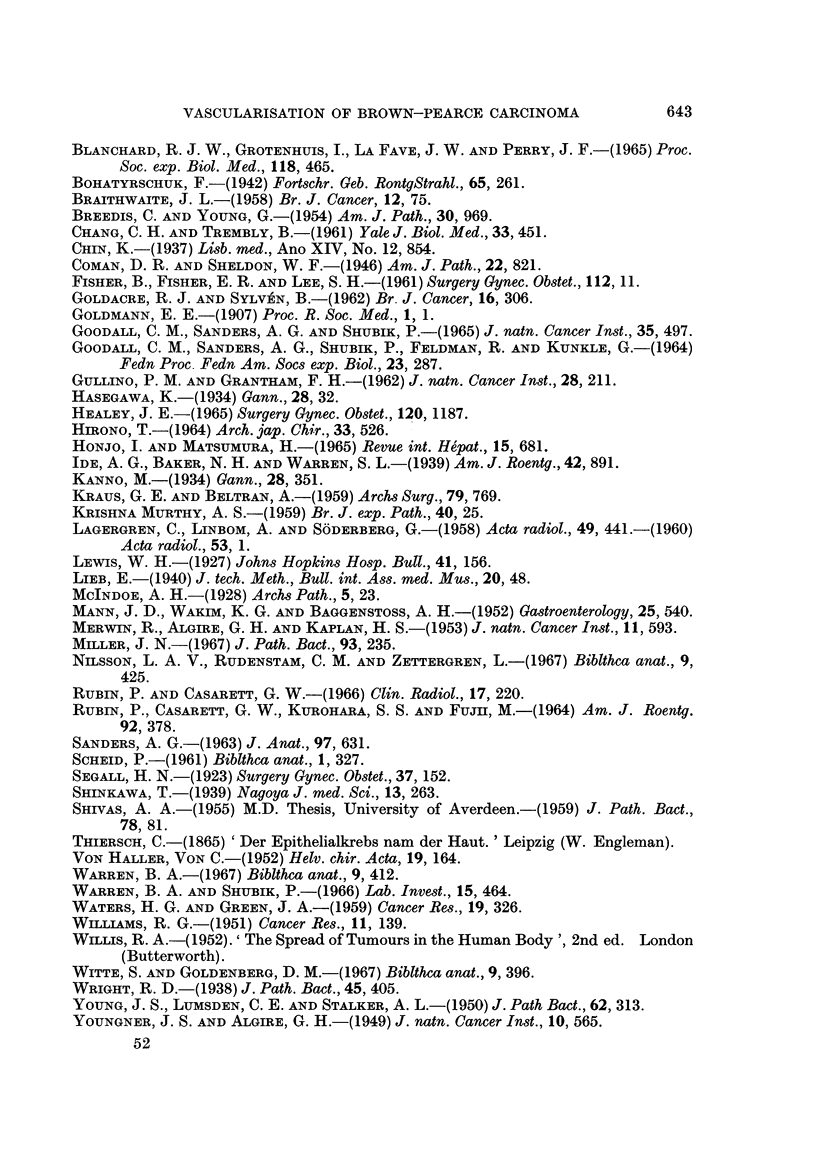# The vascularisation of Brown-Pearce carcinoma implanted in rabbit liver.

**DOI:** 10.1038/bjc.1969.79

**Published:** 1969-09

**Authors:** A. A. Shivas, W. J. Gillespie

## Abstract

**Images:**


### THE VASCULARISATION OF BROWN-PEARCE CARCINOMA IMPLANTED IN RABBIT LIVER

A. A. SHIVAS AND W. J. GILLESPIE

*From the Department of Pathology, University of Edinburgh*

Received for publication June 16, 1969

THE vasculature of tumours has been extensively studied by a variety of techniques, but interest has largely centred upon pure morphology. It is only recently that evidence has emerged for the existence of a special haemodynamic state peculiar to neoplastic growth and possibly of fundamental biological significance.

The work of Young, Lumsden and Stalker (1950), and Shivas (1959) showing the existence of a capillary and venous hypertension in Brown-Pearce carcinoma has prompted this study of its vascularisation—in liver, to determine whether it conforms to the fairly clearly defined pattern of preferential supply from the hepatic artery. The concept of neoplastic haemodynamics confers a new importance upon the positive results obtained, and the findings of earlier workers must be re-examined.

The present study utilised an injection technique with neoprene latex, which possesses good power of penetration of the microcirculation (Lieb, 1940) and produces a three-dimensional cast easily studied under the dissecting microscope.

### MATERIAL AND METHODS

Healthy rabbits of mixed strains, aged 6–8 months, and weighing 2–3 kilogrammes, were used. Under nembutal anaesthesia, supplemented with ether as necessary, laparotomy was performed through a short upper midline incision and fresh fragments of Brown-Pearce carcinoma from a donor animal were implanted into the liver parenchyma using standard lumbar puncture needles. The operation was performed on 20 animals which were killed at intervals varying from 10 to 21 days after inoculation. Where a visible or palpable tumour was present the liver was removed after identification of the portal vein and hepatic artery, and perfused with normal saline through portex cannulae at pressures approximating to the normal in the living animal (130 mm. Hg in the hepatic artery, 15 cm. water in the portal vein). When the effluent from the hepatic vein ran consistently clear, coloured neoprene latex was injected under similar pressures. The cast was then fixed in a bath of concentrated hydrochloric acid for 48 hours, with a change of acid at 24 hours. Fat was then cleared by washing in 2-ethoxy-ethanol for 24 hours, and the casts were stored in distilled water with a crystal of thymol to prevent the growth of fungus. The importance of using fresh neoprene latex cannot be too strongly stressed, as prolonged storage undoubtedly diminishes the powers of penetration of the latex.

### RESULTS

Tumour growth was obtained in 8 of the 20 animals. Four livers were perfused through the portal vein, 1 through the hepatic artery and 3 through both vessels simultaneously using different colours of neoprene. The findings are summarised in Table I.

TABLE I.—*Experimental Findings*

| Animal Number | Route of injection | Findings |
|---|---|---|
| 2 | . Portal vein | . Filling defect in cast: no filling of tumour vessels. |
| 3 | . Portal vein | . Filling defect in cast: no filling of tumour vessels. |
| 4 | . Portal vein | . Filling defect in cast: no filling of tumour vessels. |
| 6 | . Portal vein/Hepatic artery | . Foci of tumour vessels filled from hepatic artery: no evidence of filling from portal vein. |
| 9 | . Portal vein/Hepatic artery | . Tumour vessels filled mainly from hepatic artery: marginal filling from portal vein. |
| 12 | . Portal vein | . Filling defect in cast: no filling of tumour vessels. |
| 16 | . Hepatic artery | . Complete filling of tumour vessels from hepatic artery: no filling from portal vein. |
| 17 | . Portal vein/Hepatic artery | . Tumour vessels filled mainly from hepatic artery: marginal filling from portal vein. |

It seems clear that the blood supply to Brown-Pearce carcinoma implanted in the rabbit liver is essentially derived from the hepatic artery when the circulation is studied 10–21 days after implantation. Perfusion through the portal vein produces a cast which shows the site of tumour growth as a " filling defect " (Fig. 1). There is, however, some evidence (Case 9) of a marginal connection with the portal circulation following simultaneous perfusion through hepatic artery and portal vein (Fig. 3). The tumour vasculature displays a characteristic appearance of twisted, strap-like vessels communicating freely to produce a rather sponge-like appearance (Fig. 2). The channels are 5–8 times the diameter of the hepatic sinusoids and smaller vessels were not observed in the tumour. Some tortuosity and dilatation of the afferent hepatic artery branch was noted.

### DISCUSSION

Thiersch (1865) displayed the blood supply of tumours by Indian ink injection, as did Goldmann (1907) when he described the spiral coiling of vessels and capillary offshoots at the margins of growing tumours, and compared the vascularity of carcinoma and sarcoma. Lewis (1927) made Indian ink injection studies of a number of rat tumours and first pointed out that each type of tumour has a characteristic vascular pattern. He wrote, " The blood vessels do not determine the growth of the tumour; but the tumour determines the growth and pattern of blood vessels ", and he speculated further on the physical properties of tumour circulation, suggesting that necrosis was caused by the collapse of vessels under a high " tissue pressure ". It is indeed remarkable that this idea was not followed up. The illogical concept of tumour " outgrowing its blood supply " remained the popular explanation of focal necrosis in tumours, up to the present time.

In the last 25 years a considerable body of published work on the topic has accumulated and is summarised in Table II. The salient points in our present state of knowledge may, however, be conveniently stated as follows:

1. Vessels within tumours appear abnormal in size, calibre and distribution, radiologically, histologically, and in tissue culture, and the differentiation into arterioles and venules seen in granulation tissue is rarely observed.

2. Large nutrient stem vessels outwith the tumour may show tortuosity and dilatation.

3. Each individual type of tumour is characterised by a particular vascular pattern.

4. The rate of growth of host vessels into implanted tumour seems faster than

TABLE II.—*Published Work on the Vascular Morphology of Tumours*

| Method of study | Author |
|---|---|
| Dye injection and histology . | Waters and Green (1959)<br>Goldacre and Sylvén (1962) |
| Angiography . . . . . | Hasegawa (1934)<br>Chin (1937)<br>Shinkawa (1939)<br>Braithwaite (1958) |
| Micro-angiography . . . . | Bohatyrschuk (1942)<br>Lagergren *et al.* (1958)<br>Lagergren *et al.* (1960)<br>Chang and Trembly (1961)<br>Rubin *et al.* (1964)<br>Rubin and Casarett (1966) |
| Histology . . . . . . | Coman and Sheldon (1946)<br>Von Haller (1952)<br>Scheid (1961) |
| Transparent chamber techniques . | Ide *et al.* (1939)<br>Algire (1943)<br>Algire and Chalkley (1945)<br>Youngner and Algire (1949)<br>Algire and Legallais (1951)<br>Williams (1951)<br>Merwin *et al.* (1953)<br>Sanders (1963)<br>Goodall *et al.* (1964)<br>Goodall *et al.* (1965)<br>Warren and Shubik (1966)<br>Warren (1967)<br>Witte and Goldenberg (1967) |

into granulation tissue, resulting in the hypothesis of a " vessel growth stimulating substance " acting upon host vascular tissues.

5. Tumour necrosis is frequently observed. This may be a result of occlusion of vessels by extravascular pressure.

6. The response of tumour parenchyma to vascular occlusion is variable.

7. Hypotension in the host peripheral circulation may cause a reduction in the vascularity of a tumour associated with necrosis of tumour cells.

Quantitative studies have been rare until recently. Young, Lumsden and Stalker (1950) demonstrated that the " tissue pressure " in Brown-Pearce carcinoma is consistently much higher than in adjacent normal tissue, and Shivas (1955) showed that this was also true in tumours grown intracerebrally, even in the presence of raised intracranial pressure. The " tissue pressure " measured by Young and his co-workers is the minimum pressure required to introduce a bland fluid into the tissue through a hollow needle. It is thus in effect also the minimum

---

EXPLANATION OF PLATES

FIG. 1.—Cast of hepatic vasculature produced by injection via the portal vein. The site of tumour growth is seen as a cavity or " filling defect ", as the injection mass fails to enter it. × 3.

FIG. 2.—Cast of hepatic vasculature produced by injection via the hepatic artery. The tumour vasculature is fully penetrated and appears as a sponge-like mesh of bizarre, strap-like vessels (centre). × 14.

FIG. 3.—Simultaneous injection via hepatic artery and portal vein was only partially successful. Tumour vessels can be seen within the cavity. Detailed study suggested some marginal filling of tumour vessels from the portal circulation. × 7.

1

2

3

hydrostatic pressure which must be exerted by a thin-walled blood vessel if it is to remain patent. These findings in Brown-Pearce carcinoma seem explicable only on the basis of a special haemodynamic state embodying capillary and venous hypertension.

The development of tumour vasculature in tissues such as liver which themselves possess a special circulation thus acquires a new importance. It has interested many workers over the last 50 years. Segall (1923) gave the first account, following injection of human livers with a barium gelatin preparation in the post mortem room. He showed in a small number of cases that the vessels in metastatic tumours filled when the hepatic artery was injected but not when the injection was made through the portal vein. McIndoe (1928), studying the vascular lesions of portal cirrhosis, noted that metastatic tumours were supplied by the hepatic artery. Since then many published reports have confirmed this finding both in human material and experimental tumours (see Table III).

TABLE III.—*Published Work Supporting Hepatic Arterial Supply to Liver Tumours*

| Neoplasms in which hepatic arterial rather than portal venous supply has been demonstrated | Source | Methods |
|---|---|---|
| Human primary hepatic neoplasms . . . . | Bierman *et al.* (1957) | Angiography |
| | Mann *et al.* (1952) | Vynyl casts |
| Human metastatic hepatic neoplasms . . . . | Segall (1923) | Gelatin casts |
| | McIndoe (1928) | Vynyl casts |
| | Wright (1938) | Gelatin casts |
| | Breedis and Young (1954) | Gelatin casts |
| | Bierman *et al.* (1957) | Angiography |
| | Healey (1965) | Vynyl casts |
| Experimental primary hepatic neoplasms . . . . | Krishna Murthy (1959) | Indian ink injection |
| | Gullino and Grantham (1962) | Vynyl casts |
| | Nilsson *et al.* (1967) | Microangiography |
| Experimental metastatic hepatic neoplasms. . . | Kanno (1934) | Angiography |
| | Kraus and Beltran (1959) | Ligation and tumour survival experiments |
| | Fisher *et al.* (1961) | Indian ink injections |
| | Hirono (1964) | Ligation and tumour survival experiments |
| | Balashev (1965) | Histology |
| | Blanchard *et al.* (1965) | Radioactive microspheres |

Honjo and Matsumara (1965), however, using carmine gelatin injection and histology, described variable findings in rat hepatic tumours. In primary hepatic tumours induced with 3-methyl DAB, they found that cholangiocarcinoma appeared to be nourished exclusively by arterial blood, but that hepatoma showed both arterial and portal blood supply. The supply to implanted Walker 256 carcinoma was primarily arterial but in marginal areas portal supply could also be demonstrated. Nilsson *et al.* (1967) found some evidence of portal supply to rat hepatoma induced in the same way.

It might be expected that the experiments in which ligation of the hepatic artery was practised would demonstrate tumour necrosis and regression when the principal blood supply was thus removed but the reports are conflicting. Nilsson *et al.* (1967) found that after hepatic artery ligation the arterially supplied cholangiocellular tumours and secondary tumours in rat liver showed marked necrosis, but hepatocellular tumours with a dual supply tended to survive. Hirono (1964) found no change in the survival time, growth rate, or incidence of metastasis in a variety of tumours implanted into rat liver, when the hepatic artery was ligated 7 days later. Indeed, he claimed improved survival after ligation of the portal vein, as did Kraus and Beltran (1959) using Walker 256 carcinoma. Fisher *et al.* (1961) confirmed the arterial supply to hepatic metastases, but gave evidence of an augmented " take rate " in animals whose hepatic arteries or portal veins were ligated just before or within 72 hours after inoculation with tumour cells. These findings are difficult to explain in terms of information on the source of tumour vasculature, and it is planned to study this in a further series of experiments, examining the morphology of tumour circulation after ligation of the hepatic artery. However, it seems clear that when the choice exists, a growing metastatic tumour in the liver draws its circulation preferentially from the hepatic artery. This may be understood on the basis of the presence of a capillary venous hypertension in the tumour circulation (however it may be mediated) requiring an afferent supply of greater than portal venous pressure. The possibility of the higher oxygen tension in the hepatic arterial blood being the factor influencing the selection must be considered. Miller (1967) believes that the low oxygen tension in the liver plays a part in determining the high frequency and large size of hepatic metastases, but other factors must surely be involved. Willis (1952), commenting on the frequency of metastatic tumour in the liver, suggests that the high " nutritive " quality of the portal blood may be important in providing a favourable environment. This view if certainly not supported by existing experimental work. It may well be that the morphology of the hepatic vasculature with wide sinusoids and incomplete vessel walls, in association with a slow rate of flow are the important features. Tumour emboli may be easily arrested and less subject to early thrombus formation which renders non-viable so many potential metastases in other sites. An experiment in which the oxygen tensions in the arterial and venous supplies to the liver were reversed might further clarify the problem. Notwithstanding these hypothetical considerations, the present evidence seems to indicate that the pressure of the afferent blood supply is of importance. It may be of critical importance in the establishment of metastatic tumour generally, and further experiments in this field are planned.

### SUMMARY

1. Evidence is presented indicating that in Brown-Pearce carcinoma implanted in the liver, the tumour is nourished almost exclusively by the hepatic artery.

2. The possible significance of this finding in relation to the existence of capillary-venous hypertension in the tumour circulation is discussed.

3. The literature is briefly reviewed.

4. A programme of further experimental work is suggested.

### REFERENCES

ALGIRE, G. H.—(1943) *J. natn. Cancer Inst.*, **4**, 13.

ALGIRE, G. H. AND CHALKLEY, H. W.—(1945) *J. natn. Cancer Inst.*, **6**, 73.

ALGIRE, G. H. AND LEGALLAIS, F. Y.—(1951) *J. natn. Cancer Inst.*, **12**, 399.

BALASHEV, R. H.—(1965) *Vop. Onkol.*, **11**, 48.

BIERMAN, H. R., BYRON, R. L., KELLEY, K. H. AND GRADY, A.—(1957) *J. natn. Cancer Inst.*, **12**, 107.

BLANCHARD, R. J. W., GROTENHUIS, I., LA FAVE, J. W. AND PERRY, J. F.—(1965) *Proc. Soc. exp. Biol. Med.*, **118**, 465.
BOHATYRSCHUK, F.—(1942) *Fortschr. Geb. RontgStrahl.*, **65**, 261.
BRAITHWAITE, J. L.—(1958) *Br. J. Cancer*, **12**, 75.
BREEDIS, C. AND YOUNG, G.—(1954) *Am. J. Path.*, **30**, 969.
CHANG, C. H. AND TREMBLY, B.—(1961) *Yale J. Biol. Med.*, **33**, 451.
CHIN, K.—(1937) *Lisb. med.*, Ano XIV, No. 12, 854.
COMAN, D. R. AND SHELDON, W. F.—(1946) *Am. J. Path.*, **22**, 821.
FISHER, B., FISHER, E. R. AND LEE, S. H.—(1961) *Surgery Gynec. Obstet.*, **112**, 11.
GOLDACRE, R. J. AND SYLVÉN, B.—(1962) *Br. J. Cancer*, **16**, 306.
GOLDMANN, E. E.—(1907) *Proc. R. Soc. Med.*, **1**, 1.
GOODALL, C. M., SANDERS, A. G. AND SHUBIK, P.—(1965) *J. natn. Cancer Inst.*, **35**, 497.
GOODALL, C. M., SANDERS, A. G., SHUBIK, P., FELDMAN, R. AND KUNKLE, G.—(1964) *Fedn Proc. Fedn Am. Socs exp. Biol.*, **23**, 287.
GULLINO, P. M. AND GRANTHAM, F. H.—(1962) *J. natn. Cancer Inst.*, **28**, 211.
HASEGAWA, K.—(1934) *Gann.*, **28**, 32.
HEALEY, J. E.—(1965) *Surgery Gynec. Obstet.*, **120**, 1187.
HIRONO, T.—(1964) *Arch. jap. Chir.*, **33**, 526.
HONJO, I. AND MATSUMURA, H.—(1965) *Revue int. Hépat.*, **15**, 681.
IDE, A. G., BAKER, N. H. AND WARREN, S. L.—(1939) *Am. J. Roentg.*, **42**, 891.
KANNO, M.—(1934) *Gann.*, **28**, 351.
KRAUS, G. E. AND BELTRAN, A.—(1959) *Archs Surg.*, **79**, 769.
KRISHNA MURTHY, A. S.—(1959) *Br. J. exp. Path.*, **40**, 25.
LAGERGREN, C., LINBOM, A. AND SÖDERBERG, G.—(1958) *Acta radiol.*, **49**, 441.—(1960) *Acta radiol.*, **53**, 1.
LEWIS, W. H.—(1927) *Johns Hopkins Hosp. Bull.*, **41**, 156.
LIEB, E.—(1940) *J. tech. Meth., Bull. int. Ass. med. Mus.*, **20**, 48.
MCINDOE, A. H.—(1928) *Archs Path.*, **5**, 23.
MANN, J. D., WAKIM, K. G. AND BAGGENSTOSS, A. H.—(1952) *Gastroenterology*, **25**, 540.
MERWIN, R., ALGIRE, G. H. AND KAPLAN, H. S.—(1953) *J. natn. Cancer Inst.*, **11**, 593.
MILLER, J. N.—(1967) *J. Path. Bact.*, **93**, 235.
NILSSON, L. A. V., RUDENSTAM, C. M. AND ZETTERGREN, L.—(1967) *Biblthca anat.*, **9**, 425.
RUBIN, P. AND CASARETT, G. W.—(1966) *Clin. Radiol.*, **17**, 220.
RUBIN, P., CASARETT, G. W., KUROHARA, S. S. AND FUJII, M.—(1964) *Am. J. Roentg.* **92**, 378.
SANDERS, A. G.—(1963) *J. Anat.*, **97**, 631.
SCHEID, P.—(1961) *Biblthca anat.*, **1**, 327.
SEGALL, H. N.—(1923) *Surgery Gynec. Obstet.*, **37**, 152.
SHINKAWA, T.—(1939) *Nagoya J. med. Sci.*, **13**, 263.
SHIVAS, A. A.—(1955) M.D. Thesis, University of Averdeen.—(1959) *J. Path. Bact.*, **78**, 81.
THIERSCH, C.—(1865) ' Der Epithelialkrebs nam der Haut. ' Leipzig (W. Engleman).
VON HALLER, VON C.—(1952) *Helv. chir. Acta*, **19**, 164.
WARREN, B. A.—(1967) *Biblthca anat.*, **9**, 412.
WARREN, B. A. AND SHUBIK, P.—(1966) *Lab. Invest.*, **15**, 464.
WATERS, H. G. AND GREEN, J. A.—(1959) *Cancer Res.*, **19**, 326.
WILLIAMS, R. G.—(1951) *Cancer Res.*, **11**, 139.
WILLIS, R. A.—(1952). ' The Spread of Tumours in the Human Body ', 2nd ed. London (Butterworth).
WITTE, S. AND GOLDENBERG, D. M.—(1967) *Biblthca anat.*, **9**, 396.
WRIGHT, R. D.—(1938) *J. Path. Bact.*, **45**, 405.
YOUNG, J. S., LUMSDEN, C. E. AND STALKER, A. L.—(1950) *J. Path Bact.*, **62**, 313.
YOUNGNER, J. S. AND ALGIRE, G. H.—(1949) *J. natn. Cancer Inst.*, **10**, 565.